# Calcitonin gene-related peptide monoclonal antibodies and medication overuse headache: is stopping excessive pain medication still necessary?

**DOI:** 10.1055/s-0045-1809658

**Published:** 2025-07-28

**Authors:** João José Freitas de Carvalho

**Affiliations:** 1 Faculdade de Medicina Unichristus, Fortaleza CE, Brazil.

**Keywords:** Headache, Headache Disorders, Secondary, Migraine Disorders, Tension-Type Headache, Therapeutics, Analgesics

## Abstract

Recent studies demonstrate a significant paradigm shift concerning migraine patients suffering from medication overuse (MO). Traditionally, doctors used to demand that patients who overused medications be withdrawn before beginning any preventive therapy; however, such a belief has recently been challenged by emerging evidence about the benefit of calcitonin gene-related peptide monoclonal antibodies (CGRP mAbs), which have shown similar effectiveness in several clinical trials and real-world studies, regardless of whether a patient has previously stopped taking excessive medications. The data indicates that patients undergoing CGRP mAb therapy naturally decreased their acute medication consumption as migraine frequency diminished without requiring forced discontinuation. Furthermore, safety analyses have confirmed favorable tolerability profiles when CGRP mAbs are administered concurrently with various acute medications. This new evidence-based approach offers several clinical advantages, including enhanced treatment adherence and reduced risk of withdrawal complications. These findings support transitioning from mandatory detoxification protocols toward more individualized treatment strategies, representing a significant advancement in clinical migraine management.

## INTRODUCTION

Concomitant medication overuse (MO) and medication overuse headache (MOH) classically represent a significant challenge in migraine treatment. The management of these patients has traditionally centered on discontinuation of overused medications concurrent with initiating preventive therapy. However, the advent of calcitonin gene-related peptide monoclonal antibodies (CGRP mAbs) has challenged this established paradigm. In recent years, a growing body of evidence suggests that these innovative therapeutics can effectively treat migraine prophylaxis without requiring prior analgesic discontinuation, representing a significant shift in our approach and management. This sems to be a pharmacological class effect as randomized clinical trials (RCTs) and real-world studies demonstrated similar efficacy and tolerability of all these new medicines in chronic (CM) and episodic migraine (EM), regardless of medication overuse (MO) and various previous treatment failures. 1

## CLINICAL EFFICACY WITHOUT PRIOR DETOXIFICATION

Supporting this approach, Pensato et al. conducted a comparative study to examine, specifically, outcomes between patients who underwent detoxification and those who did not before starting CGRP mAb therapy. Prospectively, they used data from 401 patients with chronic migraine and MOHs who had utilized at least 28 days of analgesics monthly, followed for 3 months. Their results revealed equivalent efficacy between groups, providing strong evidence that prior detoxification is not essential for treatment success. 2

A multicenter study further validated this approach, showing significant improvements in chronic migraine with MOH through CGRP mAb therapy, regardless of prior detoxification status. 3 Over a 3-month follow-up period, patients exhibited a significantly greater reduction in the number of headache days as well as in symptomatic medication intake compared with the control group ( *p* < 0.0001). Their findings suggest that the traditional emphasis on medication withdrawal may be unnecessarily burdensome for patients.

## REAL-WORLD EVIDENCE

An innovative real-world analysis by Scheffler et al. demonstrated remarkable success rates in MOH resolution without mandatory detoxification. While this study was retrospective and single-center, it found that 60.6 to 89% of patients successfully transitioned from MOH to non-MOH status through CGRP mAb treatment alone. 4 This finding directly challenges the traditional requirement for medication withdrawal before preventive treatment initiation.

In 2021, Caronna et al. provided additional real-world evidence through their 6-month effectiveness study, demonstrating comparable improvement rates between medication-overuse and non-overuse populations. 5 This was a prospective study conducted in chronic migraine patients with and without medication overuse treated with monthly MAbs (erenumab/galcanezumab). This research showed that patients naturally reduced their acute medication use as their migraine frequency decreased under CGRP mAb therapy without requiring forced discontinuation.

## EARLY TREATMENT RESPONSE AND SAFETY PROFILE

Recent research by Ito et al. (2023) examined the early effects of CGRP mAbs (erenumab, galcanezumab and fremanezumab) in MOH patients, documenting significant reductions in monthly headache days as early as 1 month into treatment. 6 This rapid response occurred without mandatory analgesic discontinuation, suggesting that delayed treatment initiation for detoxification may unnecessarily prolong patient suffering.

The safety profile of this approach has been well-documented. De Luca et al., in a prospective study on oxidative stress biomarkers, conducted a comprehensive safety analysis, finding no significant increase in adverse events when CGRP mAbs were administered alongside various acute medications. 7 This favorable safety profile has been consistently reported across multiple studies, including long-term follow-up data.

## PATIENT-CENTERED BENEFITS AND MANAGEMENT STRATEGIES

Tanei and Saito (2024), aiming to evaluate the real-world clinical results of anti-calcitonin gene-related peptide monoclonal antibody (CGRP mAb) treatment for migraine with MOH without abrupt drug discontinuation and no hospitalization, demonstrated successful outpatient management of MOH using CGRP mAbs without requiring hospitalization or abrupt medication discontinuation. 8 This approach offers several advantages: a) improved treatment adherence, b) reduced impact on daily activities, c) better patient acceptance of therapy, and d) lower risk of withdrawal symptoms.

Another prospective study conducted at the University Hospital of Modena, provided evidence that even severely impaired patients can benefit from direct CGRP mAb initiation. 9 Their work emphasized the importance of individualized treatment approaches rather than rigid adherence to traditional detoxification protocols.

For cases with suboptimal response, Iannone et al. (2023) developed strategies for switching between different CGRP mAbs, demonstrating that treatment can be optimized without reverting to mandatory detoxification. 10 This flexibility in management approaches allows for better tailoring of therapy to individual patient needs.

To measure treatment success, Sette et al. (2022) proposed a novel index for monitoring acute medication use during CGRP mAb therapy. This index provides a structured approach to tracking treatment success without requiring complete medication discontinuation. 11 This tool helps clinicians make informed decisions about treatment continuation and modification.

## UNDERSTANDING SUSTAINED EFFICACY DURING MEDICATION OVERUSE

Koumprentziotis and Mitsikostas (2022) explored the mechanistic basis for CGRP mAb effectiveness in MOH, explaining why these treatments can succeed without prior detoxification. 12 Their work highlights how the specific targeting of the CGRP pathway remains effective despite ongoing medication use.

## GUIDELINES AND CLINICAL IMPLEMENTATION

The European Academy of Neurology's guidelines on MOH management have begun to acknowledge the changing landscape of treatment options. 13 While not altogether abandoning the concept of medication withdrawal, these guidelines recognize the potential for alternative approaches, particularly with newer therapeutic options like CGRP mAbs.

Multiple observational studies provide additional support for this approach. Curone et al. (2020) 14 and Di Cola et al. (2020) 15 documented successful outcomes in real-world settings without mandatory detoxification, providing practical evidence for the feasibility of this approach.

In conclusion, the recent cumulative evidence strongly supports the position that discontinuation of overused analgesics is not necessary before initiating CGRP mAb therapy for migraine prophylaxis.

As demonstrated, multiple lines of evidence support this conclusion:

- Equivalent efficacy in patients with and without prior detoxification;
- Favorable safety profile regardless of concurrent medication use;
- Improved patient acceptance and treatment adherence;
- Natural reduction in acute medication use following successful treatment;
- Practical advantages in real-world clinical settings.

This paradigm shift represents a significant advancement in migraine management, offering a more patient-friendly approach without compromising treatment outcomes. The success of CGRP mAbs in treating migraine with concurrent medication overuse suggests that we should move away from rigid detoxification requirements and toward more flexible, individualized treatment approaches.
